# The British Congress of Tuberculosis

**Published:** 1901-08

**Authors:** 


### The British Congress of Tuberculosis.

The British Congress of Tuberculosis for the Prevention of Consumption, was opened in London, July 22, 1901, by Field-Marshal H.R.H., the Duke of Cambridge, president of the congress, who welcomed the foreign delegates and spoke of the interest taken in the congress by King Edward. Lord Lansdowne, the Foreign Secretary, also welcomed the delegates and in the name of the government promised all the assistance the government was able to afford the congress in its endeavors to combat a disease more desolating than war.

After the Lord Mayor of London, Frank Green, Lord Strathcona and Mount Royal and others had addressed the congress, Lord Lister, one of the British vice-presidents of the congress, in a few words conveyed the thanks of that body to Prof. Robert Koch, of Germany, and the other scientists present for their work, saying they knew the enemy they had to deal with and that it was not only the prevention, but the cure of consumption, that the congress hoped to effect.

A telegram was read from King Edward to the Duke of Cambridge, as follows: "I pray you heartily to welcome for me the eminent men of almost every nation who have assembled under your presidency and to express to them my earnest hope that the result of the deliberations of the congress will be to assist the world in mitigating this dire disease, which has baffled the most distinguished physicians for so long."

Prior to the adjournment of the first session the Duke of Cambridge announced that a gift of £120,000 would be forthcoming for the purpose of establishing the first public tuberculosis sanitarium, as soon as the recommendations of the congress concerning its establishment had been formulated.

Four hundred foreign delegates attended the opening session. They included a number of Americans and Canadians. Several ambassadors and foreign ministers, including Joseph H. Choate, the American Ambassador, also were present.

Mr. I. N. Ford, special London correspondent of the New York *Tribune* said, under date of July 24, Robert Koch's address before the congress was an event of world-wide importance, since he was the recognised leader in the campaign against consumption. The hall was crowded with medical men, and there was a group of scientific experts around Lord Lister on the platform. Professor Koch was introduced by Lord Lister with the simplicity becoming to each great man of science, and was welcomed with British heartiness. His address occupied about eighty minutes, and was followed with intense interest. Professor Koch read it in English, with a deliberate painstaking effort to repress his marked German accent, and with no lack of emphasis when controversial passages were reached.

Tall, full habited, with a high forehead, large spectacles and stooping shoulders, he was the embodiment of German scholarship and thoroughness in scientific investigation.

Professor Koch's main theme was the best method of fighting tuberculosis in the light of experience gained in combating the bubonic plague, cholera, hydrophobia and especially leprosy, which he described as caused by a parasite closely resembling the tubercle bacillus. He pronounced hereditary consumption to be extremely rare, and considered that the sputum of a consumptive patient was the chief source of infection.

He gave an account of recent experiments at Berlin which served to prove that human tuberculosis could not be transferred to animals. Lord Lister subsequently admitted that the evidence seemed satisfactory. Koch had also satisfied himself that the converse proposition was also true, and that human beings were not susceptible to bovine tuberculosis communicated through milk, butter and meat. This conclusion Lord Lister was unwilling to accept on the evidence cited by Koch, and several experts from the continent thrashed out the matter with various results. Koch himself declared that the danger of infection by the milk and flesh of tubercular cattle was hardly greater than by hereditary transmission, and that measures against it were inadvisable. The chief source of human tuberculosis was the diffusion of sputum, and natural preventive measures were the removal of patients from small, overcrowded dwellings, the establishment of special hospitals for them, compulsory notification to the health authorities of cases of tubercular disease, systematic disinfection of sick rooms and the founding of sanatoriums where cures could be effected.

Koch took a hopeful view of both preventive and curative measures, explained how much good work had been done by consumptive hospitals in England, and highly praised Dr. Biggs's system of organisation in New York as worthy of the study and imitation of all municipal and sanitary authorities.

